# Large-scale lipidomics profiling reveals characteristic lipid signatures associated with an increased cardiovascular risk

**DOI:** 10.1007/s00392-023-02260-x

**Published:** 2023-07-20

**Authors:** Tobias Harm, Kristina Dittrich, Adrian Brun, Xiaoqing Fu, Moritz Frey, Alvaro Petersen Uribe, Frederic-Joaquim Schwarz, Anne-Katrin Rohlfing, Tatsiana Castor, Tobias Geisler, Dominik Rath, Michael Lämmerhofer, Meinrad P. Gawaz

**Affiliations:** 1 Department of Cardiology and Angiology, University Hospital Tübingen, Eberhard Karls University Tübingen, Otfried-Müller-Straße 10, 72076 Tübingen, Germany; 2 Institute of Pharmaceutical Sciences, Eberhard Karls University Tübingen, Auf der Morgenstelle 8, 72076 Tübingen, Germany

**Keywords:** Platelets, Lipidome, Coronary artery disease, Cardiovascular risk, Bleeding

## Abstract

**Background and aims:**

Patients with cardiovascular disease (CVD) are at high risk to develop adverse events. The distinct risk of developing adverse cardiovascular (CV) events is not solely explained by traditional risk factors. Platelets are essentially involved in progression of CVD including coronary artery disease (CAD) and platelet hyperreactivity leads to development of adverse CV events. Alterations in the platelet lipidome lead to platelet hyperresponsiveness and thus might alter the individual risk profile. In this study, we investigate the platelet lipidome of CAD patients by untargeted lipidomics and elucidate alterations in the lipid composition of patients with adverse CV events.

**Methods:**

We characterized the platelet lipidome in a large consecutive CAD cohort (*n* = 1057) by an untargeted lipidomics approach using liquid chromatography coupled to mass spectrometry.

**Results:**

The platelet lipidome in this study identified 767 lipids and characteristic changes occurred in patients with adverse CV events. The most prominent upregulated lipids in patients with cardiovascular events primarily belong to the class of phospholipids and fatty acyls. Further, upregulated platelet lipids are associated with an increased cardiovascular or bleeding risk and independently associated with adverse events. In addition, alterations of the platelet lipidome are associated with modulation of in vitro platelet functions.

**Conclusions:**

Our results reveal that the composition of the platelet lipidome is altered in CVD patients with an increased cardiovascular risk and distinct platelet lipids may indicate adverse events. Results of this study may contribute to improved risk discrimination and classification for cardiovascular events in patients with CVD.

**Graphical abstract:**

Main findings of this study and hypothetical impact of altered platelet lipid signatures in patients with adverse cardiovascular events on platelet function and clinical outcome. *LPE* lysophosphatidylethanolamines, *CAR* acylcarnitines, *FA* fatty acids.

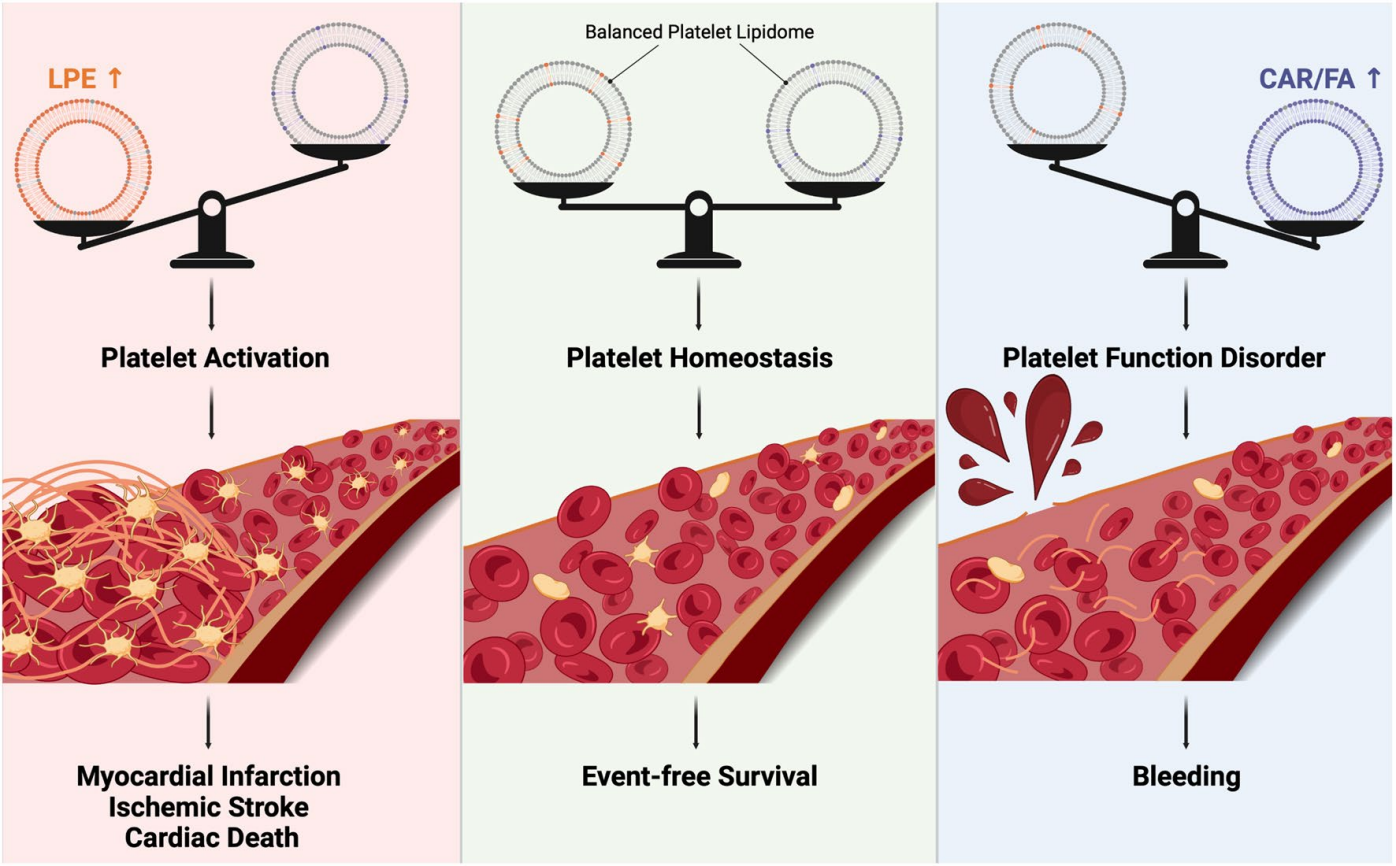

## Introduction

Platelets play a critical role in haemostasis and atherothrombosis [1]. Platelet activation and hyperreactivity is associated with poor clinical outcome in patients with symptomatic coronary artery disease (CAD) [2–4]. At site of a vulnerable plaque, platelet adhesion and aggregation trigger intracoronary thrombus formation, leading to myocardial ischaemia and acute coronary syndrome (ACS) [5]. Beyond their critical role in thrombosis, platelets have been recognized to govern mechanisms of thrombo-inflammation and immuno-thrombosis, mechanisms involved in progression of CAD [6–8]. A sustained hyperreactivity of circulating platelets is associated with adverse thrombo-ischaemic events in both chronic (CCS) and acute coronary syndrome (ACS) [9].

Dyslipoproteinaemia is a major cardiovascular risk factor that determines the clinical course of CAD [10, 11]. We and others found that enhanced levels of lipoproteins, such as low-density lipoprotein (LDL) promote platelet activation and thrombo-inflammation [12–14]. Increased uptake of oxidized LDL enhances platelet activation and substantially modulates the platelet lipidome [12]. Further, significant alterations of the platelet lipidome occur in patients with ACS, which may contribute to the pathophysiology of the disease [15, 16]. The assessment of distinct molecular lipid signatures in patients with CAD reflects therapeutic effects of lipid-lowering treatment and outperforms conventional measurements of lipoproteins [17].

In this study, we performed platelet lipidome profiling with mass spectrometry in a large-scale (*n* > 1000) prospective cohort study enrolling patients with symptomatic CAD and analysed the association of 767 distinct lipid species with platelet function and cardiovascular risk over 3 years after study enrolment.

## Methods

### Study population

One-thousand and fifty-seven (1057) patients were enrolled in this prospective, consecutive study (Table 1). All patients underwent catheter angiography within 24 h after hospital admission and were treated for symptomatic CAD. Blood sampling was performed according to a standardized protocol from peripheral venipuncture with patients fasting overnight for 12 h. Platelet isolation and preparation for mass spectrometry analysis was performed as previously described [15, 16].

**Table 1:** Baseline characteristics of patient population

	All	No adverse events	Adverse events	*p* value
	(*n* = 1057)	(*n* = 985; 93.2%)	(*n* = 72; 6.8%)	
Female, *n* (%)	318 (30.1)	303 (30.8)	15 (20.8)	0.067
Age, years (mean ± SD)	70.2 (± 11.6)	69.9 (± 11.5)	74.2 (± 12)	**0.004**
Body mass index (mean ± SD)	27.5 (± 5.1)	27.6 (± 5.1)	27.0 (± 4.6)	0.366
Cardiovascular risk factors				
Arterial hypertension, n (%)	945 (89.4)	879 (89.1)	66 (91.7)	0.518
Hyperlipidemia, *n* (%)	815 (77.1)	757 (76.9)	58 (80.6)	0.470
Diabetes mellitus, *n* (%)	319 (30.2)	294 (29.9)	25 (34.7)	0.384
Current smoking, *n* (%)	197 (18.6)	187 (18.9)	10 (13.9)	0.280
Ex smoking > 6 mo, *n* (%)	228 (21.6)	208 (21.1)	20 (27.8)	0.188
Obesity, *n* (%)	257 (24.3)	238 (24.2)	19 (26.4)	0.671
Atrial fibrillation, *n* (%)	246 (23.3)	223 (22.6)	23 (31.9)	0.073
Previous CABG, *n* (%)	47 (4.5)	40 (4.1)	4 (9.7)	**0.025**
Previous MI, *n* (%)	226 (21.4)	207 (21)	19 (26.4)	0.283
Renal function (GFR) (mean ± SD)	80.1 (± 40.7)	80.4 (± 41.4)	75.9 (± 29.5)	0.232
Medication on admission				
Statins, *n* (%)	855 (80.1)	795 (80.7)	60 (83.3)	0.521
Acetylsalicylic acid, *n* (%)	931 (88.1)	871 (88.4)	60 (83.3)	0.198
Clopidogrel, *n* (%)	460 (43.5)	432 (43.9)	28 (38.9)	0.429
Ticagrelor, *n* (%)	223 (21.1)	216 (21.9)	17 (23.6)	0.726
Prasugrel, *n* (%)	157 (14.9)	149 (15.1)	8 (11.1)	0.359
Cangrelor, *n* (%)	1 (0.1)	1 (0.1)	0 (0)	0.787
Oral anticoagulants, *n* (%)	232 (22)	210 (21.3)	22 (30.6)	0.068
Angiotensin-converting enzyme inhibitors, *n* (%)	373 (35.3)	349 (35.4)	24 (33.3)	0.740
Angiotensin II receptor antagonists, *n* (%)	498 (47.1)	465 (47.2)	33 (45.8)	0.850
Angiotensin receptor-neprilysin inhibitor, *n* (%)	12 (1.4)	11 (1.1)	1 (1.4)	0.718
Aldosterone antagonists, *n* (%)	239 (22.6)	219 (22.5)	20 (27.8)	0.267
SGLT-2 inhibitors, *n* (%)	60 (5.7)	56 (5.7)	4 (5.6)	0.765
Ca channel antagonists, *n* (%)	369 (34.9)	344 (34.9)	25 (34.7)	0.995
β-Blockers, *n* (%)	720 (68.1)	663 (67.3)	57 (79.2)	0.053
Diuretics, *n* (%)	377 (35.7)	347 (35.2)	30 (41.7)	0.259
Lipid profile parameters				
LDL-cholesterol (mg/dL) (mean ± SD)	106 (± 44.3)	108.1 (± 60.4)	105.9 (± 43.1)	0.775
HDL-cholesterol (mg/dL) (mean ± SD)	46.8 (± 19.8)	46.8 (± 20)	45.6 (± 15.5)	0.541
Triglycerides (mg/dL) (mean ± SD)	145.7 (± 108.3)	147.3 (± 110.5)	121.1 (± 63.6)	**0.004**
Total cholesterol (mg/mL) (mean ± SD)	165.7 (± 46.4)	165.7 (± 44.9)	165.2 (± 65.2)	0.954
Platelets (mean ± SD)	226.5 (± 67.6)	226.5 (± 66.5)	225.8 (± 81)	0.944
Disease				
Chronic coronary syndrome, *n* (%)	396 (37.5)	371 (37.7)	25 (24.5)	0.619
Unstable angina, *n* (%)	192 (18.2)	185 (18.8)	7 (9.7)	0.054
NSTEMI, *n* (%)	326 (30.8)	295 (30)	31 (43.1)	**0.020**
STEMI, *n* (%)	93 (8.8)	87 (8.8)	6 (8.3)	0.884
Peripheral artery disease, *n* (%)	50 (4.7)	47 (4.8)	3 (4.2)	0.815

Normally distributed data were analysed using Student’s t-test. Non-normally distributed data were compared using the Mann–Whitney U test. Mean values are presented as mean ± standard deviation (SD) and significant baseline characteristics (*p* < 0.05) are highlighted

All patients completed a standardized questionnaire on smoking status, previous dietary factors and medication history. Further, medication on admission was assessed and patients received antiplatelet therapy and statin treatment according to current CAD guidelines and disease severity. Cardiovascular risk factors were assessed according to the Framingham score and outlined in the supplementary methods section. A clinical follow-up of cardiovascular events was performed over a 3-year period after hospital discharge and relied on a review of medical records including documentation of hospital or general practitioners. Alongside major bleeding events, the composite cardiovascular endpoint included cardiac death, myocardial infarction and ischaemic stroke. Bleeding events were categorized according to ISTH score as defined in the supplementary methods section. The study was approved by the local ethics committee of Tübingen (270/2011B01) and all patients gave written informed consent. The experiments were performed in accordance with the highest ethical standards and comply with the Declaration of Helsinki.

### Platelet lipidomics

Preparation of platelets and analysis by mass spectrometry was performed as previously described [15] and summarized in the supplementary methods section.

### Platelet function analysis

Analysis of platelet function was performed using whole blood impedance aggregometry (Multiplate) after stimulation with collagen, arachidonic acid, and ADP as previously described [9].

### Statistical analysis

Patients baseline characteristics data and pre-processed lipidomics data were analysed using JMP^®^ Version 16.2 (SAS Institute, Cary, North Carolina, USA). Normally distributed data were analysed using Student’s *t* test, non-normally distributed data were compared using the Mann–Whitney *U* test. Mean values are presented as mean ± standard deviation (SD) or as median and interquartile range (IQR) where applicable. Categorical parameters were compared using Chi-square. Orthogonal partial least square discriminant analysis (OPLS-DA) and principal component analysis (PCA) of all identified lipids were performed after normalization and boxplots of the first principal components were displayed with Mann–Whitney *U* test. Concentrations of platelet lipids were analysed by Student’s *t* test and to account for the multiple hypothesis testing, a false discovery rate (FDR) controlling procedure was further adopted to correct significance levels (*p* < 0.05) for FDR ≤ 5%. Therefore, Benjamini–Hochberg procedure (BH) was implemented for testing of significant alterations between groups. For subgroup comparison, patients were grouped into quartiles and dichotomized according to mean platelet concentrations, where applicable. Significantly altered lipids were further analysed by Cox proportional hazards models to assess the relationship of distinct lipids with the cardiovascular risk. Therefore, hazards ratios were tested for a 1-standard deviation (SD) higher lipid concentration by analysing the significance level of the correlation coefficient between Kaplan–Meier transformed survival time and scaled Schoenfeld residuals. Adjustment for age, gender and statin medication was performed. *L*_1_-regularized Cox regression with a least absolute shrinkage and selection operator (LASSO) algorithm was implemented for lipid selection and optimal *λ* was chosen by tenfold cross-validation and repeated with variant partitions to account for the potential variance of cross-validation. Likewise, adjustment for age, gender and statin medication was implemented. Further assessment of the association of selected lipids with an increased cardiovascular risk was performed by calculating Harrell’s concordance index (*C* index). Estimators of incremental predictive values and the referring confidence intervals were computed with cross-validation and percentile bootstrap analysis. PCA, OPLS-DA, Cox proportional hazards models, LASSO and C index were computed using different software packages in RStudio (RStudio Inc., Boston, USA) and are further elucidated in the supplementary method section. Correlation data are based on Pearson’s product-moment correlation coefficient (*r*) and Spearman’s rank correlation coefficient (*R*). Graphic output was performed with different software packages including RStudio and JMP.

## Results

### Platelet lipidome profiling in CAD patients by untargeted liquid chromatography–mass spectrometry

Previously, we characterized changes of the platelet lipidome in patients with coronary artery disease (CAD) and showed that significant alterations occur in patients with acute coronary syndrome (ACS) when compared to chronic coronary syndrome (CCS) or healthy controls [12, 15]. In the present study, we prospectively analysed the platelet lipidome in a large cohort of patients with symptomatic CAD utilizing an untargeted UHPLC–ESI-QTOF-MS/MS approach to elucidate associations of distinct lipids with an increased cardiovascular risk. Baseline demographic, clinical and laboratory characteristics of the 1057 patients are shown in Table 1. Over a median follow-up period of 36 months of 1057 patients (2987 person-years), 72 individuals (6.8%) experienced a major cardiovascular (cardiac death, ischaemic stroke, myocardial infarction) or bleeding event corresponding to an incidence rate of 24.1 (95% CI 18.9–30.4) per 1000 person-years **(**Table 2**).**

**Table 2:** Clinical endpoints at 3-year follow-up

36 months follow-up	Ischaemic endpoint (*n* = 51)	Bleeding endpoint (*n* = 21)
Cardiac death, *n* (%)	17 (33.3)	
Myocardial infarction, *n* (%)	21 (41.2)	
Stroke, *n* (%)	13 (25.5)	
Major bleeding, *n* (%)		21 (100)

**Fig. 1:**
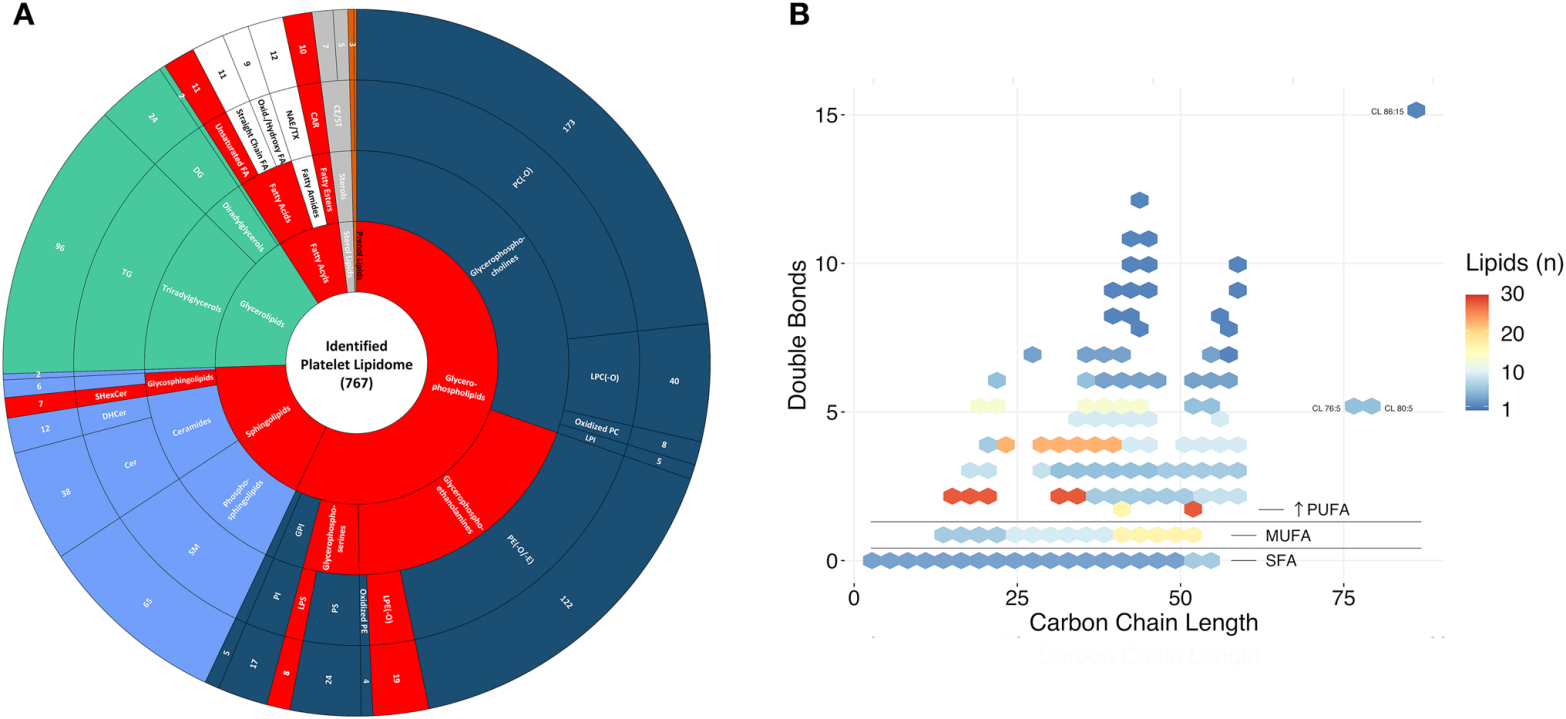
Assessment of the human platelet lipidome in the prospective CAD patient cohort reveals distinct lipid signatures. **A** The platelet lipidome in this study comprised 767 lipids from isolated circulating platelets. The lipids are attributable to 40 subclasses and six main lipid categories: glycerophospholipids (55.8% of verified lipids), sphingolipids (17.1%), glycerolipids (15.9%), fatty acyls (9.2%), sterol (1.6%) and prenol (0.4%) lipids. Platelet lipid categories are coloured accordingly and significantly altered species among patients with adverse cardiovascular events are highlighted (red). **B** Hexagonal bin plot of structural lipid analysis. The entirety of platelet lipids is depicted according to number of double bonds (*Y*-axis) and fatty acid side chain length (*X*-axis) and quantity of distinct lipid species is coloured accordingly. A predominance of lipids with carbon chain length between 25 and 45 with mainly poly-unsaturated fatty acid (PUFA) sidechain and less monounsaturated (MUFA) or saturated sidechains (SFA) is highlighted

After pre-processing and structural annotation of the lipids detected by MS, we could verify 767 lipids from isolated circulating platelets (Fig. 1A). The identified platelet lipidome of this study comprised lipids attributable to 40 subclasses, which can be summarized into six main lipid categories: glycerophospholipids (55.8% of verified lipids), glycerolipids (15.9%), fatty acyls (9.2%), sphingolipids (17.1%), sterol (1.6%) and prenol (0.4%) lipids. Further structural analysis of the platelet lipid composition highlighted a predominance of lipids with carbon chain length between 25 and 45 with mainly poly-unsaturated fatty acid (PUFA) sidechain and less monounsaturated (MUFA) or saturated (SFA) sidechains (Fig. 1B).

**Fig. 2:**
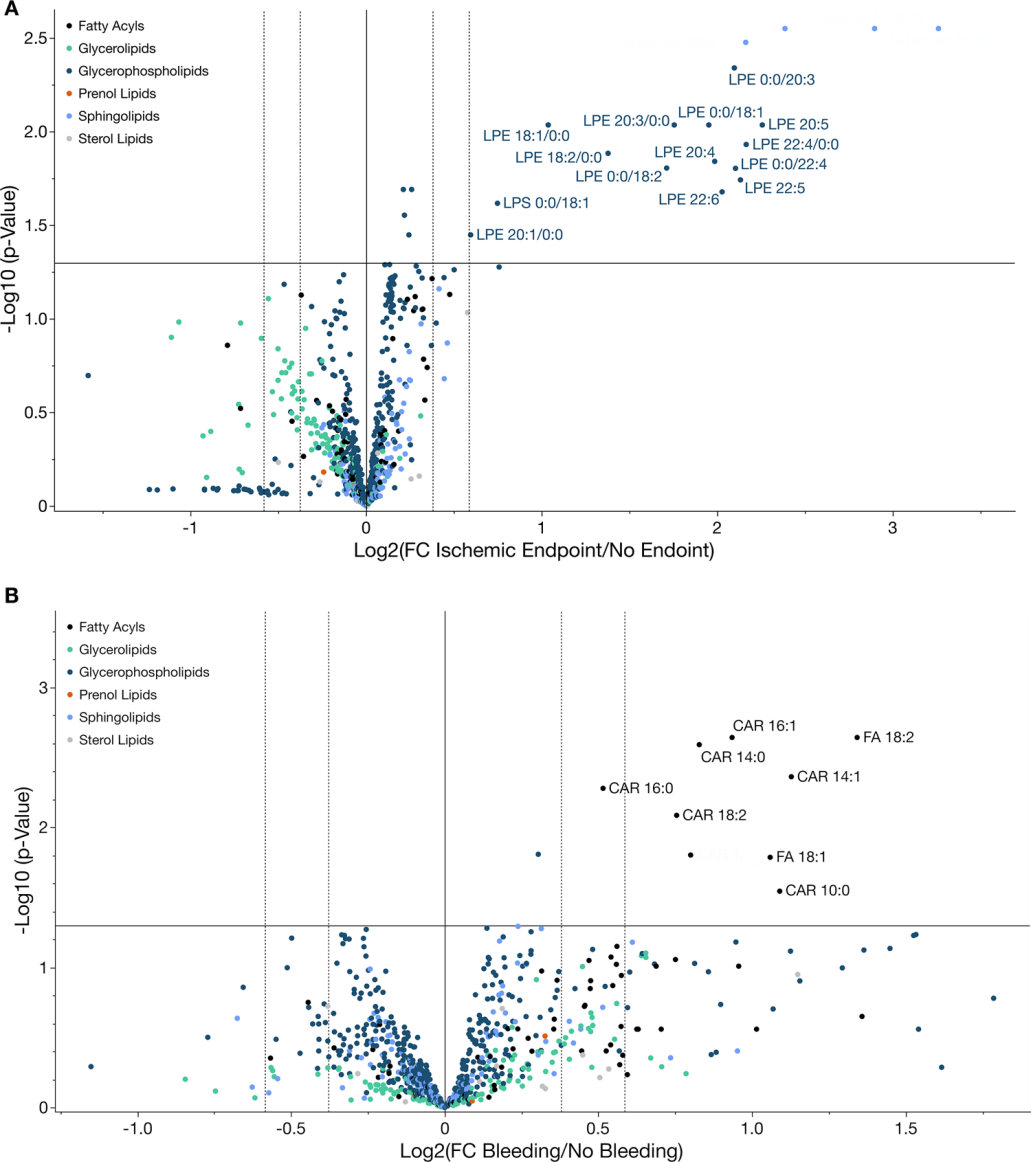
Characteristic changes in the platelet lipidome occur in patients with adverse cardiovascular events. **A** Volcano plot of all identified platelet lipids in patients with CAD. The *X*-axis (fold change, FC) displays the base 2 logarithm of the concentration ratios between patients with cardiovascular events including cardiac death, myocardial infarction or stroke (ischaemic endpoint) to those without adverse events during the 3-year follow-up (ischaemic endpoint *n* = 51, no endpoint *n* = 1006). Values > 1 indicate upregulation in patients with cardiovascular events; < 1 downregulation compared to patients without incident CVD. Y-axis showing negative log10-transformed *p* values (BH *p* < 0.05). Significantly upregulated lipids with a > 1.5-fold change are labelled and mainly comprised lysophosphatidylethanolamines and lysophosphatidylserines. **B** Volcano plot of all identified platelet lipids comparing patients with major bleeding events (*n* = 21) to those without bleeding (*n* = 1036) during the follow-up period. Significantly (BH *p* < 0.05) upregulated lipids with a > 1.5-fold increase in patients with a major bleeding are displayed and exclusively belong to the category of fatty acyls

To elucidate characteristic changes in the platelet lipidome associated with cardiovascular events, we compared the mean of relative concentrations of distinct lipids and their referring lipid subclasses between patients with and without adverse cardiovascular events during the follow-up period. We found that 18 lipids were significantly (*p* < 0.05, FDR < 5%) upregulated in patients with adverse cardiovascular events compared to patients without events (Fig. 2A). Significantly upregulated lipids mostly comprised lipids belonging to the category of glycerophospholipids [lysophosphatidylethanolamines (LPEs, *n* = 13; LPE 0:0/18:1, LPE 0:0/18:2, LPE 0:0/20:3, LPE 0:0/22:4, LPE 18:1/0:0, LPE 18:2/0:0, LPE 20:1/0:0, LPE 20:3/0:0, LPE 20:4, LPE 20:5, LPE 22:4/0:0, LPE 22:5, LPE 22:6), lysophosphatidylserine (LPS, *n* = 1; LPS 0:0/18:1), phosphocholine (PC, *n* = 3; PC 18:1–20:1, PCO-16:0–20:1, PCO-36:2) and phosphoethanolamine (PE, *n* = 1; PE 16:0–18:1)] (Fig. 2A). Interestingly, LPE concentrations were significantly downregulated in patients with statin treatment in contrast to statin-naïve patients (Supplementary Fig. S7).

A different platelet lipidome signature was found when patients were categorized into bleeding versus no bleeding within the follow-up period. Nine platelet lipids were significantly (*p* < 0.05, FDR < 5%) increased in patients with a major bleeding event during the follow-up when compared to patients free from bleeding **(**Fig. 2B**)**. Among upregulated lipids, different lipid groups comprised mainly fatty acyls [(acyl carnitines (CAR, *n* = 7, CAR 10:0, CAR 12:0, CAR 14:0, CAR 14:1, CAR 16:0, CAR 16:1, CAR 18:2), fatty acids (FA, *n* = 2, FA 18:1, FA 18:2)] and one phosphocholine (PC 18:0–22:5) **(**Fig. 2B**)**. Here, it was striking that upregulated platelet acylcarnitines in patients with bleeding events were associated with an increased bleeding risk according to PRECISE-DAPT score. The established score of predictive bleeding risk was significantly higher in patients with adverse events. We found that the number of patients with high CAR concentration was significantly increased in patients with high (61.9%) when compared to moderate (48.5%), low (46%) or very low (39.1%) PRECISE-DAPT score (Supplementary Fig. S8).

Metabolic pathway analysis of altered lipids revealed enrichment of fatty acyl and phospholipids metabolism in patients with adverse CV events when compared to patients without events during the follow-up (Supplementary Fig. S5).

**Fig. 3:**
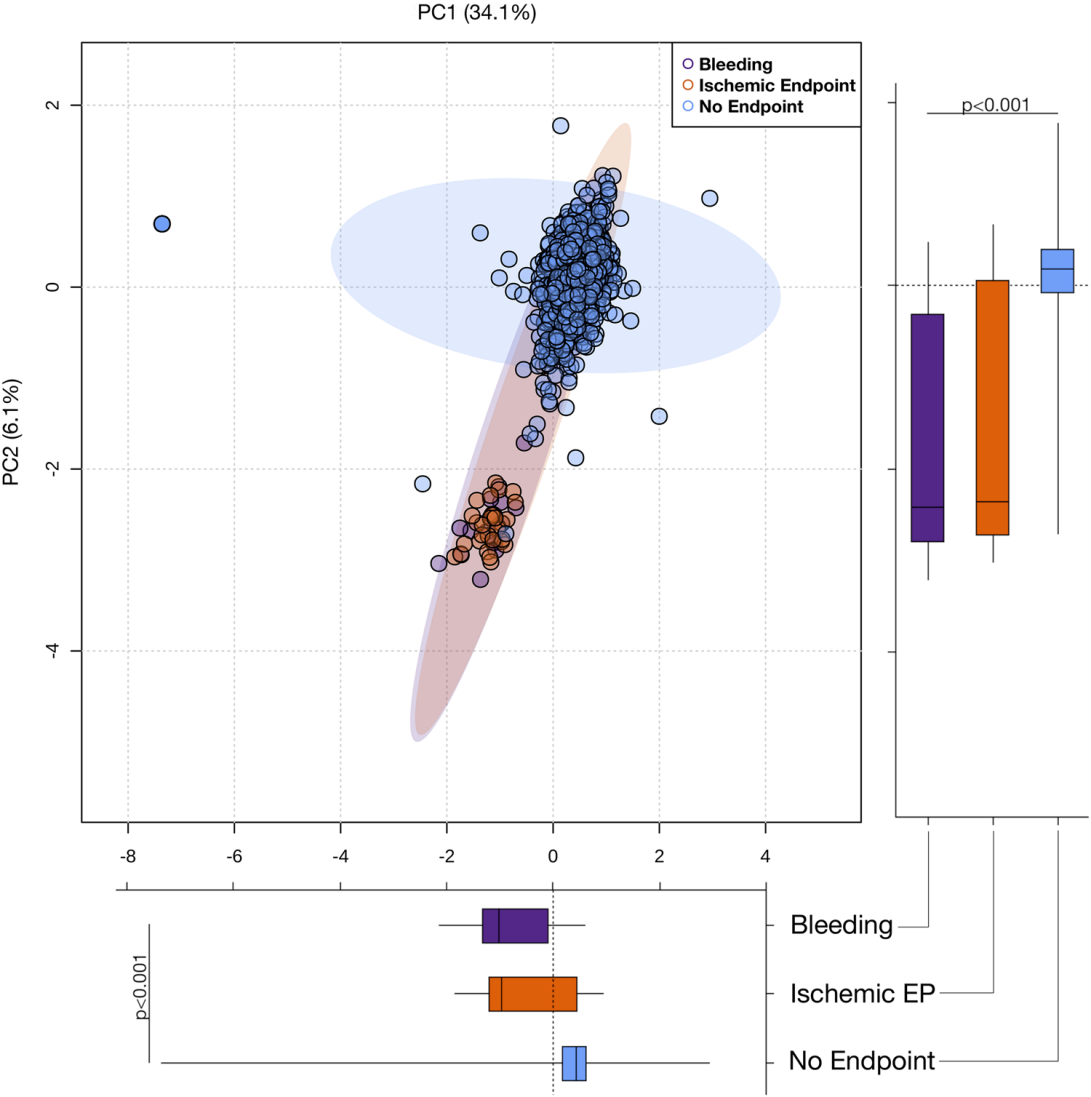
The platelet lipidome is altered in patients with adverse cardiovascular events. Principal component analysis (PCA) of the platelet lipidome (767 lipids). Clustering of patients with adverse ischaemic events (orange) or bleeding events (purple) reveals a clear separation compared to patients without adverse cardiovascular events (light blue). Box plots of first two components (PC1/2) display first and third quartiles, and whiskers extend from each quartile to the minimum or maximum values. Data were based on normalized concentrations of all identified lipids and Mann–Whitney U-test was used to analyse significant differences between cardiovascular endpoints

**Fig. 4:**
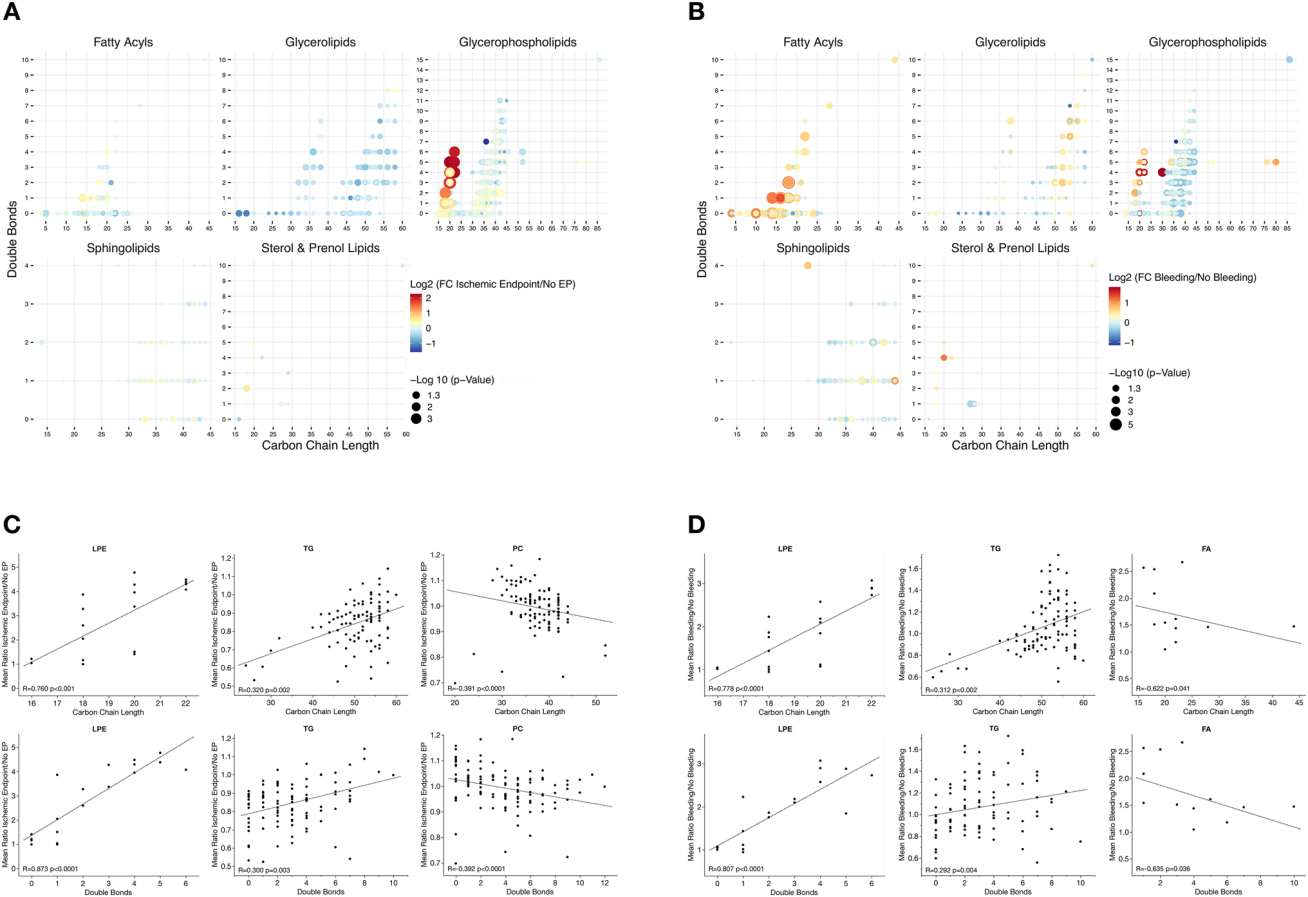
Physicochemical attributes of platelet lipid species vary among patients with adverse cardiovascular events. **A** Dot plot of identified lipids in patients with symptomatic CAD. Each dot represents a unique lipid species. *X*-axis represents fatty acyl carbon chain number and y-axis the number of double bonds. Size is scaled by negative Log-transformed p-value of comparison between lipid levels in patients with and without cardiovascular events. Colour continuously represents fold change between patients subgroups. Upregulation of glycerophospholipids mainly with low fatty acyl carbon number (*C* < 25) and poly-unsaturated side chain (DB ≥ 2) is observed in patients with cardiovascular endpoints compared to those without incident CVD. **B** The right plot depicts upregulation of mainly fatty acyls and glycerophospholipids in patients with major bleeding in contrast to those without bleeding events. **C** Mean ratio of platelet lipid abundance between patients with cardiovascular events compared to patients without adverse events by fatty acid side chain length and degree of saturation in different lipid classes. Significant (*p* < 0.05) Spearman correlations (*R*_s_) are shown. Ratio > 1 indicates a higher abundance in patients with adverse cardiovascular events. **D** Spearman correlations (*R*_s_) of lipid abundance in patients with bleeding events and degree of saturation and side chain length

To evaluate, whether critical changes in the total lipid composition of the platelet lipidome occur between patients with recurring adverse events and patients without CVD events, we performed principal component analysis (PCA) including all 767 detected lipids of this study. Clustering of patients enrolled into the 3-year follow-up showed a distinct separation of patients with adverse cardiovascular and bleeding events when compared to patients free from recurring adverse events (Fig. 3). Supervised orthogonal partial least square discriminant analysis (OPLS-DA) revealed a clear separation of patients with ischaemic events in contrast to bleeding events (Supplementary Fig. S1). Further, a distinct separation of patients with ischaemic or bleeding events in contrast to patients with an event-free follow-up is depicted in Supplementary Figs. S2 and S3. This indicates that the platelet lipidome signature in patients with adverse ischaemic and bleeding events significantly differs from patients without cardiovascular events. Remarkably, concentrations of poly-unsaturated lysoglycerophospholipids (PUFA-LPLs) were increased in patients with recurrent adverse events, whereas glycerolipids and especially triglycerides were decreased by trend in contrast to patients without CVD events (Fig. 4A). Likewise, besides mono- and poly-unsaturated fatty acyls, PUFA-PLs were increased in platelets of patients with major bleeding events. Interestingly, physiochemical attributes of distinct lipid classes significantly differed among patients with recurring cardiovascular events when compared to patients without any incident CVD during the follow-up (Fig. 4B). Side chain length (*R* = 0.760, *p* < 0.001) and degree of saturation (*R* = 0.873, *p* < 0.0001) of LPEs were significantly associated with a higher abundance in patients with recurrent cardiovascular adverse events when compared to patients without incidence of CVD events. Similar trends were observed for TG side chain length (*R* = 0.320, *p* = 0.002) and number of double bonds (*R* = 0.300, *p* = 0.003). In contrast, an inverse correlation of mean ratios between patients with recurrent cardiovascular events compared to the event-free group was shown for PC side chain length (*R* = − 0.391, *p* < 0.0001) and number of double bonds (*R* = − 0.392, *p* < 0.001) (Fig. 4C). Likewise, side chain length (*R* = 0.778, *p* < 0.0001) and degree of saturation (*R* = 0.807, *p* < 0.0001) of LPEs were associated with major bleeding events during the follow-up period. Similar results were achieved for TG side chain length (*R* = 0.312, *p* = 0.002) and double bonds (*R* = 0.292, *p* = 0.004). In contrast, FA side chain length (*R* = − 0.622, *p* = 0.041) and number of double bonds (*R* = − 0.635, *p* = 0.036) were inversely associated with major bleeding events (Fig. 4D).

### Association of adverse cardiovascular events with alterations in the platelet lipidome

**Fig. 5:**
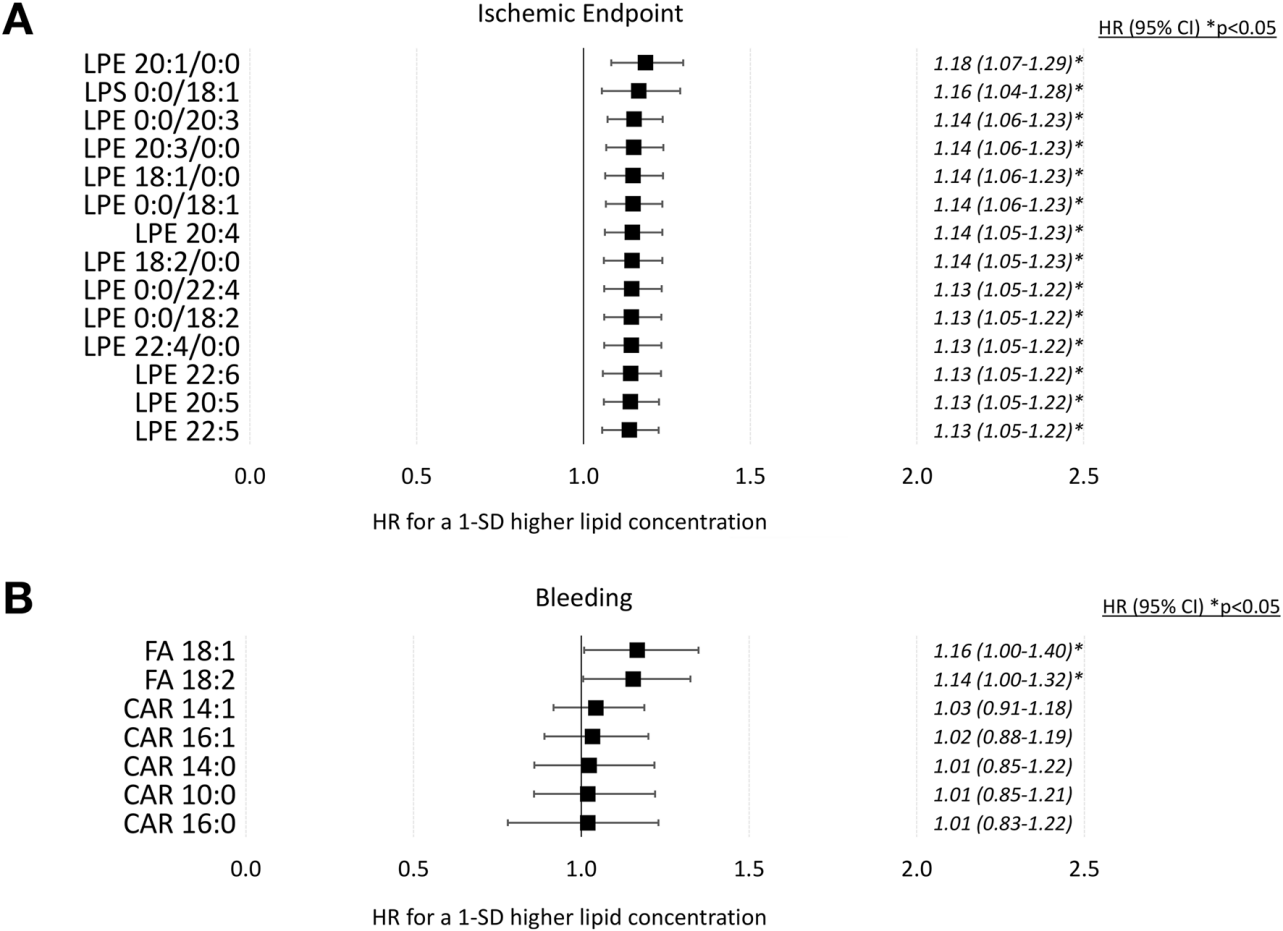
Association of distinct platelet lipid signatures with an increased hazard of developing adverse cardiovascular events. **A** Forest plot of the estimated regression coefficient (95% CI) from Cox proportional hazard models on the association of significantly upregulated lipids in patients with ischaemic endpoints and the cardiovascular risk. All lipids were found to be independently associated with an increased cardiovascular risk and hazard ratios (HR) were calculated for a 1-SD unit higher lipid concentration and adjusted for age, gender and statin treatment. Thus, especially lysophosphatidylethanolamines might be independently suitable to predict the cardiovascular risk in patients with CVD. **B** Associations of significantly upregulated platelet lipids in patients with an increased bleeding risk. High levels of acylcarnitines and fatty acids FA18:1 and FA 18:2 might indicate an increased hazard to develop major bleeding events in patients with CVD

To further elucidate the impact of changes in the platelet lipidome in patients with adverse cardiovascular events, we employed hazard models for assessing the ability of significantly altered lipids to indicate an enhanced risk for adverse cardiovascular events. Therefore, Cox proportional hazard models were calculated with 14 lipids found to be highly increased in patients with adverse cardiovascular events after adjustment for age, gender and statin treatment (Fig. 5). Upregulated lipids of patients with recurrent CVD events shared a hazard ration > 1, indicating an increased cardiovascular risk. Furthermore, lysophospholipids (LPE/LPS) were significantly associated with an increase in CV risk. Thus, LPE 20:1/0:0 (HR 1.18, 95% CI 1.07–1.29), LPE 0:0/18:1 (HR 1.16, 95% CI 1.04–1.28), LPE 0:0/20:3 (HR 1.14, 95% CI 1.06–1.23), LPE 20:3/0:0 (HR 1.14, 95% CI 1.06–1.23), LPE 18:1/0:0 (HR 1.14, 95% CI 1.06–1.23), LPE 0:0/18:1 (HR 1.14, 95% CI 1.06–1.23), LPE 20:4 (HR 1.14, 95% CI 1.05–1.23), LPE 18:2/0:0 (HR 1.14, 95% CI 1.05–1.23), LPE 0:0/22:4 (HR 1.13, 95% CI 1.05–1.22), LPE 0:0/18:2 (HR 1.13, 95% CI 1.05–1.22), LPE 22:4/0:0 (HR 1.13, 95% CI 1.05–1.22), LPE 22:6 (HR 1.13, 95% CI 1.05–1.22), LPE 20:5 (HR 1.13, 95% CI 1.05–1.22) and LPE 22:5 (HR 1.13, 95% CI 1.05–1.22) independently predicted an enhanced risk for adverse cardiovascular events in this study (Fig. 5A). Correspondingly, upregulated lipids of patients with major bleeding events indicated an increased risk of incident major haemorrhage. FA 18:1 (HR 1.16, 95% CI 1.00–1.40) and FA 18:2 (HR 1.16, 95% CI 1.00–1.40), both independently predicted an enhanced risk for major bleeding events during the follow-up period (Fig. 5B).

In conclusion, it becomes evident that distinct lipid clusters including LPE and fatty acids might be suitable to predict the cardiovascular risk in patients with CAD.

### Extraction of platelet lipid signatures to predict CV risk in patients with CAD

In order to identify new biomarkers of the platelet lipidome in patients with CAD, being eligible to sufficiently determine the risk of adverse cardiovascular events, we applied a penalized model including all lipids. Thus, overfitting of lipids is minimized and thus variable selection and regularization is optimized leading to an enhanced prediction accuracy and interpretability in the setting of omics data and generation of new potential biomarkers.

Therefore, cross-validated LASSO selection algorithm was performed and adjusted for age, gender and statin therapy. In the *L*_1_-regularized Cox regression analysis nine lipids shared nonzero coefficients and thus were related to incident CVD (Table 3A). Selected lipids mostly comprised characteristic glycerophospholipids (PC 34:2;O, LPE 20:3/0:0, PI 18-1:20:4, PE O 16-1-18:2, PI 16:0–20:4), as well as two fatty acyls (FA 18:2;2O, FA18-1) and triglycerides (TG 14:0–16:0–18:1, TG 16:0–14:1–18:1). It was further noticeable, that these lipids shared at least one of the following characteristics: (i) FA 18:1 or FA 18:2 constituent; (ii) lipid oxidation or alkenyl substituent; (iii) FA 20:3 or 20:4 fatty acid side chain.

**Table 3:** Improved assessment of the 3-year cardiovascular risk in patient with CAD by platelet lipid species

A	
Lipid	LASSO regression coefficient
PC 34:2;O	− 1591.97
LPE 20:3–0:0	− 433.13
FA 18:2;2O	− 364.01
PI 18:1–20:4	− 50.52
FA18:1	− 32.59
TG 14:0–16:0–18:1	30.46
PE O-16:1–18:2	68.048
TG 16:0–14:1–18:1	124.07
PI 16:0–20:4	371.35

B	
Model	Cross-validated *C* index (95% CI)
Conventional risk factors*	0.644 (0.612–0.741)
+ PC 34:2;O + LPE 20:3–0:0 + PI 16:0–20:4	0.650 (0.623–0.754)
Above + FA 18:2;2O + TG 16:0–14:1–18:1 + PE O-16:1–18:2	0.739 (0.703–0.816)
Above + PI 18:1–20:4 + FA18:1 + TG 14:0–16:0–18:1	0.740 (0.710–0.820)
Conventional risk factors*	
+ 3 Lipids replacing HDL and total cholesterol	0.634 (0.606–0.731)
+ 6 Lipids replacing HDL and total cholesterol	0.738 (0.698–0.810)
+ 9 Lipids replacing HDL and total cholesterol	0.741 (0.695–0.812)

(A) LASSO: All 767 lipids were standardized and adjustment for age, gender, and statin medication was implemented. L_1_-regularized Cox regression with partial likelihood cross-validation was performed and nine lipid species shared a nonzero coefficient. *LASSO* least absolute shrinkage and selection operator, *PC* phosphatidylcholine, *LPE* lysophosphatidylethanolamine, *FA* fatty acid, *PI* phosphatidylinositol, *PE* phosphatidylethanolamine, *TG* triacylglycerol

(B) Risk prediction of 1057 patients with 72 adverse cardiovascular events during the 3-year follow-up. 3 lipids include PC 34:2;O + LPE 20:3–0:0 + PI 16:0–20:4; and 6 lipids, PC 34:2;O + LPE 20:3–0:0 + PI 16:0–20:4 + FA 18:2;2O + TG 16:0–14:1–18:1 + PE O-16:1–18:2. Harrell’s concordance index (*C* Index) and confidence intervals are displayed as mean of 1000 bootstrap repetitions of fivefold cross-validation; *HDL* high-density lipoprotein. *Models considering the standard Framingham Risk Score items including age, gender, diabetes mellitus, smoking status, arterial hypertension, total cholesterol, and HDL cholesterol

### Risk stratification in CAD patients integrating platelet lipid signatures

In this study, we performed a cross-validated approach of increasing the predictive value to discriminate the risk of adverse events in patients with CAD. Thus, we calculated concordance indices of conventional cardiovascular risk factors belonging to the Framingham risk score to discriminate the 3-year CV risk and added stepwise models including selected lipids of LASSO analysis (Table 3B). The addition of three lipids with highest *ß*-coefficients PC 34:2; O, LPE 20:3/0:0 and PI 16:0–20:4 to the baseline model (*C* index 0.647, 95% CI 0.612–0.741) including conventional risk measures (including age, gender, diabetes mellitus, smoking status, arterial hypertension, total cholesterol, and HDL cholesterol) increased the *C* index by 0.006 (*C* index 0.650, 95% CI 0.623–0.754). However, an even stronger elevation of C index by 0.095 was achieved by adding six lipids including FA 18:2; 2O, TG 16:0–14:1–18:1 and PE O 16-1-18:2 (*C* index 0.739, 95% CI 0.703–0.816). Ultimately, the total cluster of nine lipids with addition of PI 18:1:20:4, FA18:1 and TG 14:0–16:0–18:1 only showed a marginal augmentation of *C* index by 0.001 (*C* index 0.740, 95% CI 0.710–0.820). To further elucidate the weight of adding new lipid biomarkers to established lipids risk measures, we replaced total cholesterol (TC) and high-density lipoprotein (HDL) with platelet lipid models. Here we found that replacing three lipids did not increase predictive value (*C* index 0.634, 95% CI 0.606–0.731), but six lipids were outperforming established measures by amplifying *C* index by 0.094 (*C* index 0.738, 95% CI 0.698–0.810). In addition, replacement of HDL and TC by all selected lipids caused the highest enhancement of *C* index by 0.097 (*C* index 0.741, 95% CI 0.695–0.812). Thus, addition of platelet lipid signatures to established cardiovascular risk factors might significantly augment the 3-year risk discrimination in patients with CAD.

### Effect of lipids associated with an increased CVD risk on platelet function

**Fig. 6:**
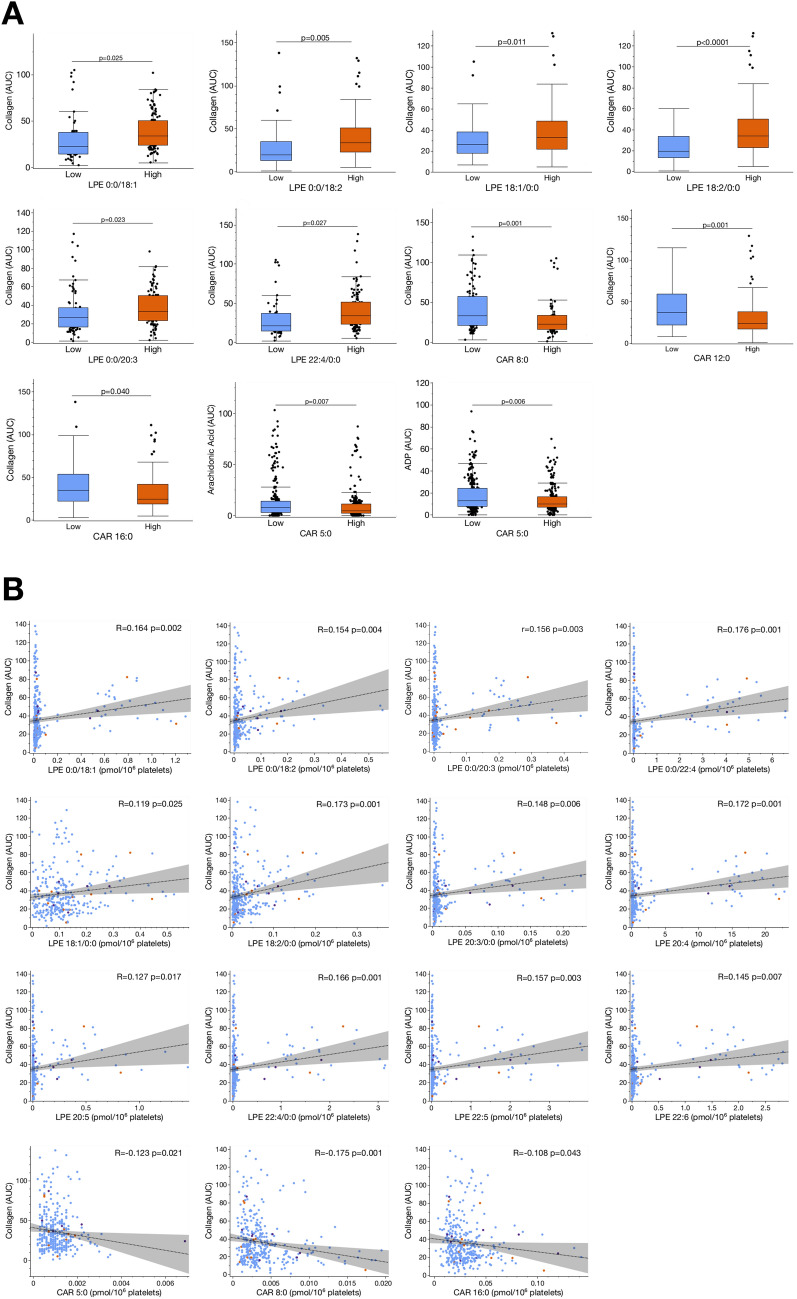
Alterations of the platelet lipidome in patients with adverse cardiovascular events modulate platelet functions and thus, might influence disease progression and outcome of patients with incident CVD. **A** Patients were divided into quartiles (upper quartile = high/lower quartile = low) according to the concentration of distinct lipids found to be significantly upregulated in patients with cardiovascular events when compared to those without adverse events. We found, that collagen-mediated platelet aggregation was significantly (Student’s *t* test, *p* < 0.05) increased under high concentrations of LPE. In contrast, high concentrations of acylcarnitines were found to be significantly (*p* < 0.05) associated with a decrease of collagen, arachidonic acid and ADP-mediated platelet aggregation. **B** Correlation of lipids found to be significantly increased in patients with cardiovascular or bleeding events compared to patients without adverse events. Collagen-mediated platelet aggregation (AUC) significantly (*p* < 0.05) correlated with increased concentrations of LPE. In contrast, collagen-mediated platelet aggregation was inversely associated with enhanced concentrations of acylcarnitines

Platelet hyperreactivity is associated with CAD and predicts outcome in CVD patients [3, 9, 18, 19]. Only recently, we found that changes in the platelet lipidome have a significant impact on platelet function [15]. To elucidate whether described changes of the platelet lipidome in patients with adverse cardiovascular events are associated with modulation of platelet function, we performed ex vivo function assays at the time of blood sampling. Patients were divided into quartiles according to the relative concentration levels of distinct lipids found to be significantly upregulated in patients with recurring adverse events when compared to those “event-free” patients. Collagen-induced platelet aggregation was significantly increased in patients with high levels of LPE 0:0/18:1 (*p* = 0.025), LPE 0:0/18:2 (*p* = 0.005), LPE 18:1/0:0 (*p* = 0.011), LPE 18:2/0:0 (*p* < 0.0001), LPE 0:0/20:3 (*p* = 0.023) and LPE 22:4/0:0 (*p* = 0.027) (Fig. 6A). In contrast, high concentrations of CAR 5:0 were associated with lowered arachidonic acid- (*p* = 0.007) and ADP-mediated (*p* = 0.006) platelet aggregation. Likewise, collagen-induced platelet aggregation was decreased in patients with high concentrations of CAR 8:0 (*p* = 0.001) and CAR 16:0 (*p* = 0.040) (Fig. 6A).

**Fig. 7:**
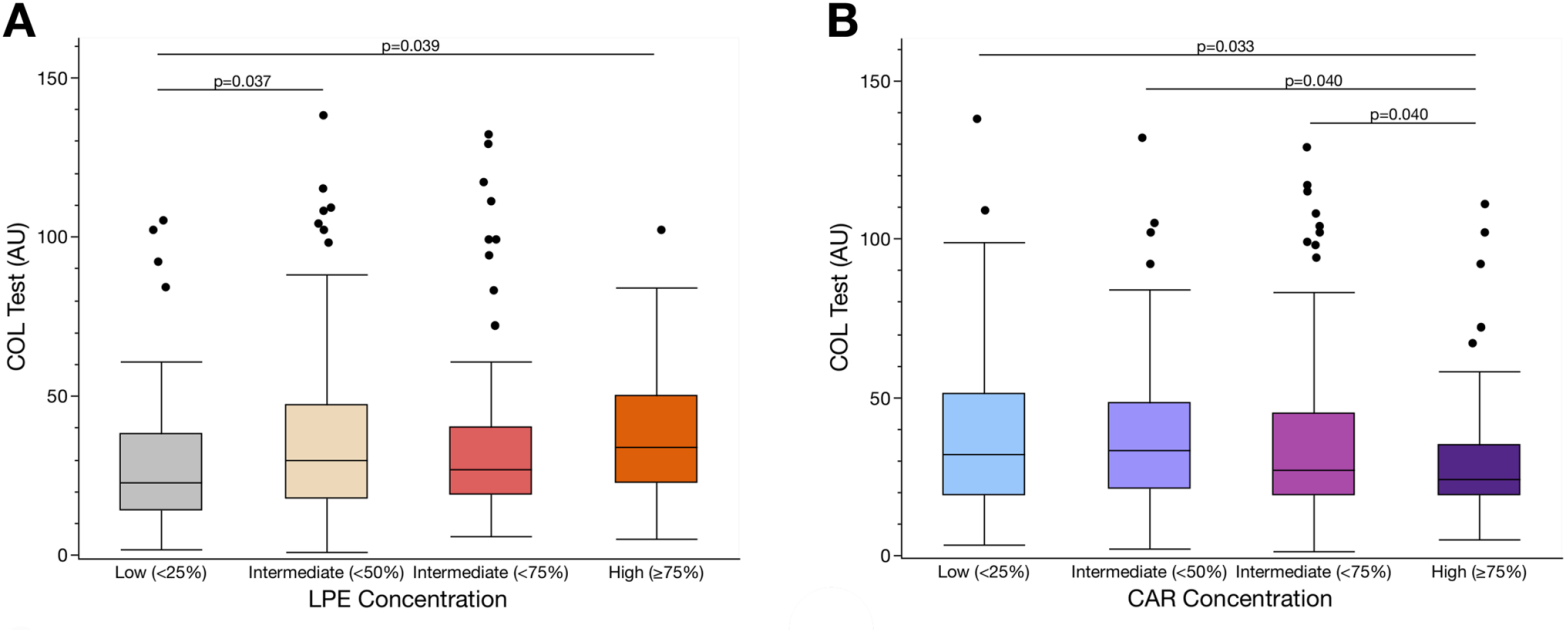
Platelet functions are altered in patients with distinct changes in the platelet lipidome. **A** The lipid class of lysophosphatidylethanolmines (LPE) was summarized by mean concentrations. All patients were divided into quartiles according to the LPE concentration (1st quartile < 25%, 2nd quartile < 50%, 3rd quartile < 75%, 4th quartile ≥ 75%). We found that collagen-mediated platelet aggregation was significantly (Student’s *t*-test, *p* < 0.05) increased under high (≥ 75%) and intermediate (< 50%) concentrations of LPE in contrast to low (< 25%) concentrations. **B** In contrast, collagen-mediated platelet aggregation was significantly (Student’s *t* test, *p* < 0.05) reduced under high (≥ 75%) concentrations of acylcarnitines (CAR) in contrast to intermediate (< 75%, < 50%) and low (< 25%) concentrations

Further correlation analysis revealed that CAR 5:0 (*r* = − 0.123, *p* = 0.021), CAR 8:0 (*r* = − 0.175, *p* = 0.001) and CAR 16:0 (*r* = − 0.108, *p* = 0.043) concentrations were inversely associated with collagen-induced platelet aggregation (Fig. 6B). In contrast, collagen-mediated platelet aggregation significantly correlated with increasing concentrations of LPE 0:0/18:1 (*r* = 0.164, *p* = 0.002), LPE 0:0/18:2 (*r* = 0.154, *p* = 0.004), LPE 0:0/20:3 (*r* = 0.156, *p* = 0.003), LPE 0:0/22:4 (*r* = 0.176, *p* = 0.001), LPE 18:1/0:0 (*r* = 0.119, *p* = 0.025), LPE 18:2/0:0 (*r* = 0.173, *p* = 0.001), LPE 20:3/0:0 (*r* = 0.148, *p* = 0.006), LPE 20:4 (*r* = 0.172, *p* = 0.001), LPE 20:5 (*r* = 0.127, *p* = 0.017), LPE 22:4/0:0 (*r* = 0.166, *p* = 0.001), LPE 22:5 (*r* = 0.157, *p* = 0.003) and LPE 22:6 (*r* = 0.145, *p* = 0.007) (Fig. 6B). Thus, high levels of lysophospholipids seem to promote platelet activation, whereas acylcarnitines lower platelet hyperreactivity in patients with CVD (Fig. 7A, B).

## Discussion

The major findings of the present study are: (1) changes of the platelet lipidome in patients with coronary artery disease are associated with adverse cardiovascular events; (2) a distinct platelet lipidome signature is associated with ischaemic and bleeding events; (3) changes of the platelet lipidome are associated with platelet function. Thus, it is tempting to speculate that assessment of distinct platelet lipids may improve evaluation of CAD patients at risk, improve the diagnostic value of conventional lipid measurement and provide new diagnostic and treatment perspectives.

Previously, substantial changes in the platelet lipidome of patients with CAD have been described [12, 15]. The platelet lipid signature is associated with disease severity and distinct platelet lipids are upregulated in ACS when compared to CCS or healthy controls [15]. Changes in the platelet lipidome occur upon activation and promote enhanced ex vivo platelet functions [20–22]. Beyond conventional cardiovascular risk factors, plasmatic lipoproteins such as low-density lipoprotein (LDL) are strongly associated with poor prognosis in CAD [23, 24]. Lowering LDL plasma levels has significantly improved the clinical outcome of CAD patients [25, 26].

Recently, platelets have been recognized as a prominent compartment of lipid metabolism and both statins and PCSK9-inhibitors modulate the platelet lipidome signature [16, 27]. However, large-scale clinical studies that investigate the platelet lipidome in CAD and its association to adverse CV events are missing so far.

In this prospective study, we characterized the platelet lipidome in a consecutive cohort of patients with symptomatic CAD and further elucidated changes of distinct platelet lipids between patients with adverse CV events and patients without incident CV events.

We found that among 767 structurally annotated lipids, 18 lipids were significantly upregulated in patients with adverse CV events. Most of the lipids were upregulated in comparison to patients without recurrence of CV events. We found that distinct fingerprints of the platelet lipidome are associated with ischaemic and bleeding events. In patients with ischaemic events glycerophospholipids (lysophosphatidylethanolamine, lysophosphatidylserine and phosphatidylethanolamine) with mono- (MUFA) or poly-unsaturated fatty acyl (PUFA) side chains were significantly upregulated compared to individuals with an event-free follow-up. PUFA-phospholipids are important metabolites in thrombo-inflammatory cascades and regulate membrane integrity and thus, essentially modulate platelet function [28, 29]. Especially PUFA-PLs with eicosatetraenoic (20:4) and docosatetraenoic acid (22:4) are proinflammatory and proapoptotic [30–32]. In our study, LPE 22:4/0:0 was significantly increased in patients with recurrence of CV events. Lysophosphatidylethanolamines promote platelet hyperreactivity [33]. In the present study we found that ex vivo platelet aggregation is enhanced in patients with high LPE content. This indicates that LPEs play a role in favouring platelet hyperreactivity and may contribute to an enhanced risk for thromboischemic events in patients with CAD. Therefore, we hypothesize that especially patients with high platelet LPE concentrations and platelet hyperreactivity might benefit from effective antiplatelet therapy to prevent adverse thromboischemic events. Further, we depict that downregulation of LPE is accessible to high potency statin treatment as LPE concentrations were decreased in patients with statin treatment compared to statin-naïve patients in this study. Thus, beyond reduction of conventional lipid parameters, downregulation of platelet LPE through lipid-lowering therapy might serve as therapeutic target to attenuate the clinical course of CAD. Therefore, CAD patients with increased concentrations of platelet LPE might benefit from intensified patient management and a close clinical follow-up.

In patients with a major bleeding event during the follow-up period, 9 lipids were significantly increased in contrast to patients without bleeding and upregulated lipids mainly comprised fatty acyls including acylcarnitines and unsaturated fatty acids. Previously, plasma acylcarnitines were found to be elevated in patients with CAD and both lipid classes showed predictive potential for mortality or recurring myocardial infarction in patients with CAD [12, 34]. Unsaturated fatty acyls are inhibitors of platelet functions and have the potential to form conjugates with aspirin, and thus potentiate the antiplatelet therapy [35–37]. Acylcarnitines inhibit plasmatic coagulation and might act as anticoagulant lipids [38]. In our study, an increase of platelet acylcarnitines was associated with a significant reduction of ex vivo aggregation response and high bleeding risk according to PREICSE-DAPT score. Thus, distinct platelet lipids were found to be associated with functional responsibility and an increased risk for adverse cardiovascular events. Therefore, it is tempting to speculate that CAD patients with high fatty acyl concentrations including platelet CAR might benefit from short-term and less aggressive dual antiplatelet treatment following PCI to prevent major bleeding events, and thus improve the long-term clinical outcome.

In our study, we highlighted that upregulated lipids were associated with an increased cardiovascular risk and especially lysophosphatidylethanolamines were significantly linked to an increased hazard of adverse CV events, whereas fatty acyls were associated with an increased risk of bleeding. To further assess the cardiovascular risk in patients with CAD with lipid measures beyond conventional parameters such as plasma lipoproteins, we selected important lipid parameters utilizing penalized LASSO algorithm. Here we included nine lipids mostly comprising glycerophospholipids. The addition of these lipids to conventional cardiovascular risk factors including traditional lipid measures improved prediction of recurrent adverse events in patients with CAD. Further, substitution of conventional lipid measures, i.e. total cholesterol and high-density lipoprotein by platelet lipids also enhanced risk discrimination of CV events in this study. Thus, we hypothesize that assessment of distinct platelet lipid biomarkers may improve prediction of adverse cardiovascular events in patients with CAD, and thus might significantly contribute to identify patients at high risk.

In conclusion, our present study implies a diagnostic value of platelet lipidomics which may be helpful to discriminate the individual risk of patients with CAD (graphical abstract and Supplementary Figure S6).

## Data Availability

The data that support the findings of this study are available on reasonable request from the corresponding author.

## References

[1] Ruggeri ZM (2002). Platelets in atherothrombosis. Nat Med.

[2] Matetzky S, Shenkman B, Guetta V, Shechter M, Beinart R, Goldenberg I, Novikov I, Pres H, Savion N, Varon D, Hod H (2004). Clopidogrel resistance is associated with increased risk of recurrent atherothrombotic events in patients with acute myocardial infarction. Circulation.

[3] Trip MD, Cats VM, van Capelle FJL, Vreeken J (1990). Platelet hyperreactivity and prognosis in survivors of myocardial infarction. N Engl J Med.

[4] Geisler T, Zürn C, Simonenko R, Rapin M, Kraibooj H, Kilias A, Bigalke B, Stellos K, Schwab M, May AE, Herdeg C, Gawaz M (2009). Early but not late stent thrombosis is influenced by residual platelet aggregation in patients undergoing coronary interventions. Eur Heart J.

[5] Gaba P, Gersh BJ, Muller J, Narula J, Stone GW (2022). Evolving concepts of the vulnerable atherosclerotic plaque and the vulnerable patient: implications for patient care and future research. Nat Rev Cardiol.

[6] Rohlfing AK, Kolb K, Sigle M, Ziegler M, Bild A, Münzer P, Sudmann J, Dicenta V, Harm T, Manke MC, Geue S, Kremser M, Chatterjee M, Liang C, von Eysmondt H, Dandekar T, Heinzmann D, Günter M, von Ungern-Sternberg S, Büttcher M, Castor T, Mencl S, Langhauser F, Sies K, Ashour D, Beker MC, Lämmerhofer M, Autenrieth SE, Schäffer TE, Laufer S, Szklanna P, Maguire P, Heikenwalder M, Müller KAL, Hermann DM, Kilic E, Stumm R, Ramos G, Kleinschnitz C, Borst O, Langer HF, Rath D, Gawaz M (2022). ACKR3 regulates platelet activation and ischemia–reperfusion tissue injury. Nat Commun.

[7] Stark K, Massberg S (2021). Interplay between inflammation and thrombosis in cardiovascular pathology. Nat Rev Cardiol.

[8] Gawaz M, Geisler T, Borst O (2023). Current concepts and novel targets for antiplatelet therapy. Nat Rev Cardiol.

[9] Droppa M, Tschernow D, Müller KAL, Tavlaki E, Karathanos A, Stimpfle F, Schaeffeler E, Schwab M, Tolios A, Siller-Matula JM, Gawaz M, Geisler T (2015). Evaluation of clinical risk factors to predict high on-treatment platelet reactivity and outcome in patients with stable coronary artery disease (PREDICT-STABLE). PLoS ONE.

[10] Brown G, Albers JJ, Fisher LD, Schaefer SM, Lin J-T, Kaplan C, Zhao X-Q, Bisson BD, Fitzpatrick VF, Dodge HT (1990). Regression of coronary artery disease as a result of intensive lipid-lowering therapy in men with high levels of apolipoprotein B. N Engl J Med.

[11] Miller M (2009). Dyslipidemia and cardiovascular risk: the importance of early prevention. QJM.

[12] Chatterjee M, Rath D, Schlotterbeck J, Rheinlaender J, Walker-Allgaier B, Alnaggar N, Zdanyte M, Müller I, Borst O, Geisler T, Schäffer TE, Lämmerhofer M, Gawaz M (2017). Regulation of oxidized platelet lipidome: implications for coronary artery disease. Eur Heart J.

[13] Stellos K, Sauter R, Fahrleitner M, Grimm J, Stakos D, Emschermann F, Panagiota V, Gnerlich S, Perk A, Schonberger T, Bigalke B, Langer HF, Gawaz M (2012). Binding of oxidized low-density lipoprotein on circulating platelets is increased in patients with acute coronary syndromes and induces platelet adhesion to vascular wall in vivo–brief report. Arterioscler Thromb Vasc Biol.

[14] Badrnya S, Schrottmaier WC, Kral JB, Yaiw K-C, Volf I, Schabbauer G, Söderberg-Nauclér C, Assinger A (2014). Platelets mediate oxidized low-density lipoprotein-induced monocyte extravasation and foam cell formation. Arterioscler Thromb Vasc Biol.

[15] Harm T, Bild A, Dittrich K, Goldschmied A, Nestele J, Chatterjee M, Fu X, Kolb K, Castor T, Borst O, Geisler T, Rath D, LäMmerhofer M, Gawaz M (2022). Acute coronary syndrome is associated with a substantial change in the platelet lipidome. Cardiovasc Res.

[16] Harm T, Frey M, Dittrich K, Goldschmied A, Rohlfing AK, Fu X, Brun A, Castor T, Rath D, Müller K, Lammerhofer M, Gawaz M (2023). Statin treatment is associated with alterations in the platelet lipidome. Thromb Haemost.

[17] Stegemann C, Pechlaner R, Willeit P, Langley SR, Mangino M, Mayr U, Menni C, Moayyeri A, Santer P, Rungger G, Spector TD, Willeit J, Kiechl S, Mayr M (2014). Lipidomics profiling and risk of cardiovascular disease in the prospective population-based Bruneck study. Circulation.

[18] Geisler T, Langer H, Wydymus M, Göhring K, Zürn C, Bigalke B, Stellos K, May AE, Gawaz M (2006). Low response to clopidogrel is associated with cardiovascular outcome after coronary stent implantation. Eur Heart J.

[19] Gawaz M, Geisler T (2009). Platelet activity: an obstacle for successful PCI. Nat Rev Cardiol.

[20] Slatter DA, Aldrovandi M, O'Connor A, Allen SM, Brasher CJ, Murphy RC, Mecklemann S, Ravi S, Darley-Usmar V, O'Donnell VB (2016). Mapping the human platelet lipidome reveals cytosolic phospholipase A2 as a regulator of mitochondrial bioenergetics during activation. Cell Metab.

[21] Cebo M, Dittrich K, Fu X, Manke MC, Emschermann F, Rheinlaender J, von Eysmondt H, Ferreirós N, Sudman J, Witte A, Pelzl L, Borst O, Geisler T, Rath D, Bakchoul T, Gawaz M, Schäffer TE, Lämmerhofer M, Chatterjee M (2022). Platelet ACKR3/CXCR7 favors antiplatelet lipids over an atherothrombotic lipidome and regulates thromboinflammation. Blood.

[22] Schuurman AR, Léopold V, Pereverzeva L, Chouchane O, Reijnders TDY, Brabander J, Douma RA, Weeghel MV, Wever E, Schomaker BV, Vaz FM, Wiersinga WJ, Veer CV, Poll TV (2022). The platelet lipidome is altered in patients with COVID-19 and correlates with platelet reactivity. Thromb Haemost.

[23] Mortensen MB, Nordestgaard BG (2020). Elevated LDL cholesterol and increased risk of myocardial infarction and atherosclerotic cardiovascular disease in individuals aged 70–100 years: a contemporary primary prevention cohort. Lancet.

[24] Silverman MG, Ference BA, Im K, Wiviott SD, Giugliano RP, Grundy SM, Braunwald E, Sabatine MS (2016). Association between lowering LDL-C and cardiovascular risk reduction among different therapeutic interventions: a systematic review and meta-analysis. JAMA.

[25] Cannon CP, Braunwald E, McCabe CH, Rader DJ, Rouleau JL, Belder R, Joyal SV, Hill KA, Pfeffer MA, Skene AM (2004). Intensive versus moderate lipid lowering with statins after acute coronary syndromes. N Engl J Med.

[26] Sabatine MS, Giugliano RP, Keech AC, Honarpour N, Wiviott SD, Murphy SA, Kuder JF, Wang H, Liu T, Wasserman SM, Sever PS, Pedersen TR (2017). Evolocumab and clinical outcomes in patients with cardiovascular disease. N Engl J Med.

[27] Petersen-Uribe Á, Kremser M, Rohlfing A-K, Castor T, Kolb K, Dicenta V, Emschermann F, Li B, Borst O, Rath D, Müller KAL, Gawaz MP (2021). Platelet-derived PCSK9 is associated with ldl metabolism and modulates atherothrombotic mechanisms in coronary artery disease. Int J Mol Sci.

[28] McEwen BJ, Morel-Kopp MC, Chen W, Tofler GH, Ward CM (2013). Effects of omega-3 polyunsaturated fatty acids on platelet function in healthy subjects and subjects with cardiovascular disease. Semin Thromb Hemost.

[29] Lagarde M, Guichardant M, Bernoud-Hubac N, Calzada C, Véricel E (2018). Oxygenation of polyunsaturated fatty acids and oxidative stress within blood platelets. Biochim Biophys Acta BBA Mol Cell Biol Lipids.

[30] Fukui M, Kang KS, Okada K, Zhu BT (2013). EPA, an omega-3 fatty acid, induces apoptosis in human pancreatic cancer cells: role of ROS accumulation, caspase-8 activation, and autophagy induction. J Cell Biochem.

[31] Heimli H, Giske C, Naderi S, Drevon CA, Hollung K (2002). Eicosapentaenoic acid promotes apoptosis in Ramos cells via activation of caspase-3 and -9. Lipids.

[32] Sherratt S, Libby P, Bhatt DL, Dawoud H, Malinski T, Mason P (2022). Abstract WP229: eicosapentaenoic acid (EPA) modulates expression of thrombotic and metabolic proteins in brain endothelium following cytokine challenge. Stroke.

[33] Xiao H, Siddiqui RA, Al-Hassani MH, Sliva D, Kovacs RJ (2001). Phospholipids released from activated platelets improve platelet aggregation and endothelial cell migration. Platelets.

[34] Shah SH, Sun J-L, Stevens RD, Bain JR, Muehlbauer MJ, Pieper KS, Haynes C, Hauser ER, Kraus WE, Granger CB, Newgard CB, Califf RM, Newby LK (2012). Baseline metabolomic profiles predict cardiovascular events in patients at risk for coronary artery disease. Am Heart J.

[35] Roy J, Adili R, Kulmacz R, Holinstat M, Das A (2016). Development of poly unsaturated fatty acid derivatives of aspirin for inhibition of platelet function. J Pharmacol Exp Ther.

[36] Smith RD, Kelly CN, Fielding BA, Hauton D, Silva KD, Nydahl MC, Miller GJ, Williams CM (2003). Long-term monounsaturated fatty acid diets reduce platelet aggregation in healthy young subjects. Br J Nutr.

[37] Sato T, Nakao K, Hashizume T, Fujii T (1987). Inhibition of platelet aggregation by unsaturated fatty acids through interference with a thromboxane-mediated process. Biochim Biophys Acta.

[38] Deguchi H, Banerjee Y, Trauger S, Siuzdak G, Kalisiak E, Fernández JA, Hoang L, Tran M, Yegneswaran S, Elias DJ, Griffin JH (2015). Acylcarnitines are anticoagulants that inhibit factor Xa and are reduced in venous thrombosis, based on metabolomics data. Blood.

